# The prehospital management of mass casualty incidents: limitations of using START in simulations and awareness of START fusion

**DOI:** 10.1186/s13049-024-01274-2

**Published:** 2024-10-18

**Authors:** Ömerul Faruk Aydın

**Affiliations:** Department of Emergency Medicine, Faculty of Medicine, Istanbul Yeni Yüzyıl University, Istanbul, Turkey

Dear Editor,

We have read with great interest the study titled “Factors affecting the accuracy of prehospital triage application and prehospital scene time in simulated mass casualty incidents” conducted by Carenzo and colleagues [1]. We believe that this study will serve as a precursor to future research, particularly in the evaluation of prehospital triage algorithms and the timing of triage in the prehospital phase. During the catastrophic earthquake in Türkiye in 2023, which resulted in the loss of 53,000 lives, our emergency departments (EDs) were overwhelmed by chaos in the face of injured individuals. In this period, through our ED research and the algorithms developed, the aim was to shed light on the prehospital process [2–6]. Drawing from our experiences, I would like to discuss some limitations in the simulation-based study conducted by Carenzo and colleagues.

The first identified limitation is related to the acronym START, which stands for “***Simple Triage and Rapid Treatment***.” This means that START is not merely a triage method but also a Triage-Treatment fusion that includes rapid interventions for the injured [7]. In this way, START differs from the triage algorithms we use in EDs in daily practice and becomes a frequently recommended method in the prehospital phase. When evaluated from this perspective, simulation studies assessing the effectiveness of START triage should also include data on interventions performed on the injured, allowing for the measurement of both triage and intervention times within the total duration. Additionally, in studies involving START, it has been observed that limited mention is made of the interventions performed on the injured, especially when compared to another widely used Mass Casualty Incident (MCI) Triage Algorithm, SALT (Sort, Assess, Lifesaving Interventions, Treatment/Transport) [8]. This is because SALT evaluates the scene management, incorporating every assessment, intervention, action, and transport into its algorithm. In this way, both researchers and readers of the study gain awareness that MCI management is an integrated process. Similarly, Carenzo and colleagues have conducted analyses regarding the overall duration of scene management [1]. At this point, prehospital MCI studies that focus solely on the timing of triage may not fully encompass the entire prehospital process.

Another limitation is the concept of over-triage and under-triage following the use of START in simulation studies. In MCIs, the first step in START triage is the differentiation of walking patients from the red, black, and yellow categories. These patients, being able to walk, are directed to a specific area with a simple command from the first responder. The critical role of the first responder here is to distinguish between black, red, and yellow patients and to perform early interventions (such as applying a tourniquet if there is bleeding) before continuing with the triage. However, differentiating black from red and red from yellow patients in the prehospital phase is not as straightforward as it is in hospital EDs, and both under-triage and over-triage can have devastating consequences. The 83.6% accuracy reported in Carenzo’s study is therefore very significant. It should also be noted that in simulation studies, green triage patients in START are not able to gather themselves in a designated area (a process that could be referred to as “self-triage”), which may cause additional delays for the first responder in simulation studies [1].

Finally, Carenzo’s study highlights the importance of prehospital time as a valuable parameter in MCIs, which I find to be a highly positive contribution in raising awareness. This emphasis is particularly significant because, during an airplane crash in Türkiye—another major MCI—Yilmaz at al. observed in our ED that the length of the prehospital process was correlated with the trauma scores of the injured, and that prolonged time could be predictive of patient outcomes [9].

In conclusion, the study by Carenzo and colleagues is supportive and comprehensive in many aspects of previous research. The intersection of simulation studies with real disaster experiences, as demonstrated in this study, provides communities and institutions with more realistic preparation opportunities by raising awareness of potential limitations during the preparedness phase. Additionally, it can enhance awareness among first responders.

## Data Availability

No datasets were generated or analysed during the current study.
